# Simplified model for pre-code RC column exposed to fire followed by earthquake

**DOI:** 10.1038/s41598-022-13188-z

**Published:** 2022-05-28

**Authors:** Ioanna Ioannou, Tiziana Rossetto, David Rush, José Melo

**Affiliations:** 1 EPICentre, Department of Civil, Environmental and Geomatic Engineering, UCL, London, UK; 2 School of Engineering, Institute of Infrastructure and Environment, University of Edinburgh, Edinburgh, UK; 3 Faculdade de Engenharia, Universidade do Porto, Porto, Portugal

**Keywords:** Civil engineering, Natural hazards

## Abstract

The behaviour of pre-code reinforced concrete (RC) columns in sequence of fire and earthquake is not well understood and can be critical in case of buildings which experienced fire and are either unrepaired or poorly repaired when exposed to an earthquake. This study proposes a framework on how to construct a simplified model to assess the post-fire cyclic behaviour of such columns. Emphasis is given to the development of simplified material models which can be used to describe the performance of the confined concrete, as its post-fire behaviour is not well studied. The model’s performance is validated against the experimental results of a square, non-seismically designed RC column. Three scenarios are considered. The reference scenario, where the column is exposed only to cyclic loading. In the other two, the column is firstly exposed to an ISO-834 time–temperature curves in a furnace of 30 min and 90 min duration and after it cooled down, it is exposed to cyclic loading. The results showed that simplified material models can be used to capture the post-fire cyclic behaviour of an RC column, built without seismic design. It was also found that the confined model adopted played an important role after the peak strength is reached.

## Introduction

Reinforced concrete (RC) is often used for the construction of mid- and high rise- multifamily dwellings. Although this material is considered to behave well when exposed to fire, fire remains a potentially very damaging hazard for such structures. Fire, as is well known, is a frequent hazard which affects a structure by increasing the temperature of some structural elements leading to the degradation of their material properties. However, fire is not the only hazard that RC structures could experience in their lifetime. In moderate or high seismicity areas, earthquakes are also an ever present hazard. Over the life of a structure, it is possible that these two hazards can affect the structure in sequence leading to a greater overall damage than the damage caused by either single hazard. In this study, we are concerned with the sequence of fire followed by an earthquake hazard. In seismic areas, this sequence has a high likelihood. A future earthquake is therefore likely to affect RC buildings that have had previous fire exposure. Recent experimental as well as analytical studies showed the negative impact of prior fire exposure to the seismic behaviour mainly of columns^1–7^ but also of structural walls^8^ as well as a non-seismically designed frame^9^. It should be mentioned that pre-cast^4^ as well as cast-in-place columns (with ordinary^1–3,6^ as well as sustainable concrete^5^) have been considered. Two studies also examined^2,7^ the post-fire cyclic behaviour of columns strengthened with fibre reinforced polymer (FRP). Nonetheless, in the absence of a high-profile case that such sequence can significantly affect RC buildings’ performance, such a scenario is not well studied and it is not clear how it affects the overall capacity of the building. This can be critical in case of buildings which experienced fire and are either unrepaired or poorly repaired when exposed to an earthquake.

Key to the analysis of the performance RC structures subjected to a fire and earthquake hazard sequence, is the use of reliable material models which can describe the cumulative effects of the hazards on the concrete (unconfined and confined) and reinforcing steel. The relevant literature is very limited; the most relevant studies focusing on the post-fire seismic behaviour of an ordinary seismically-designed column^1^ or structural wall^8^. There are established equations for accounting for the residual degradation of unconfined concrete strength and steel yield strength under fire. However, when considering the cyclic behaviour of a fire affected RC element, concrete confinement plays an important role. A review of existing literature shows that relevant existing studies have adopted only one confinement model (i.e. Mander et al. used by^3^ and Chang and Mander adopted by^1,8^), and that there is no assessment of the influence of confinement model choice on the resulting predicted behaviour of RC elements exposed to post-fire cyclic loading.

In this study, a simplified model to assess the post-fire seismic behaviour of a RC column is developed. The proposed model aims to capture the decrease in the ductility of the RC column and its enhanced strength degradation during the horizontal cycling loading caused by the decrease in the strength of concrete and steel due to their exposure in high temperatures. Key aspect of this study is the investigation of the influence of confinement model choice on the predicted behaviour of an RC column subjected to post-fire cyclic loading. Experimental data are adopted for the performance evaluation of different confinement models, with the aim of providing guidance on this topic. Given the important role of seismic design in the performance of an RC structure during an earthquake, a non-seismically designed RC structure is considered here which concerns a large number of existing buildings and is also associated with thinner concrete cover, which constitute the main fire protection for the reinforcing steel. The simplified models developed in this study are compared against the experiments conducted as part of the Challenging Risk project in the Structural and Fire Resistance Laboratory of the Aveiro University^2^. The experiments aimed to examine the impact of fire intensity in the seismic performance of an ordinary 0.30 m square column, which was exposed to fire on all four sides. The column is designed to an old seismic code^10^ and is representative of the ground floor columns in a 4-storey RC building in the Mediterranean region.

In what follows, the framework to construct a simplified model to assess the post-fire cyclic behaviour of an RC column is described. Then the framework is applied to the pre-code RC column and the results are compared to their experimental counterparts. A detailed description of the experiments and a rigorous discussion of the results can be found in^2^, which presents them as part of a series of experiments on the post-fire cyclic behaviour of pre-code and strengthened RC columns. For this reason, in the present study the experimental design is only briefly described and the experimental results are used to validate the analytical solution developed here. The reader is advised to refer to^2^ for more information on the experimental results.

## Framework for modelling post-fire cyclic behaviour of RC column

**Figure 1:**
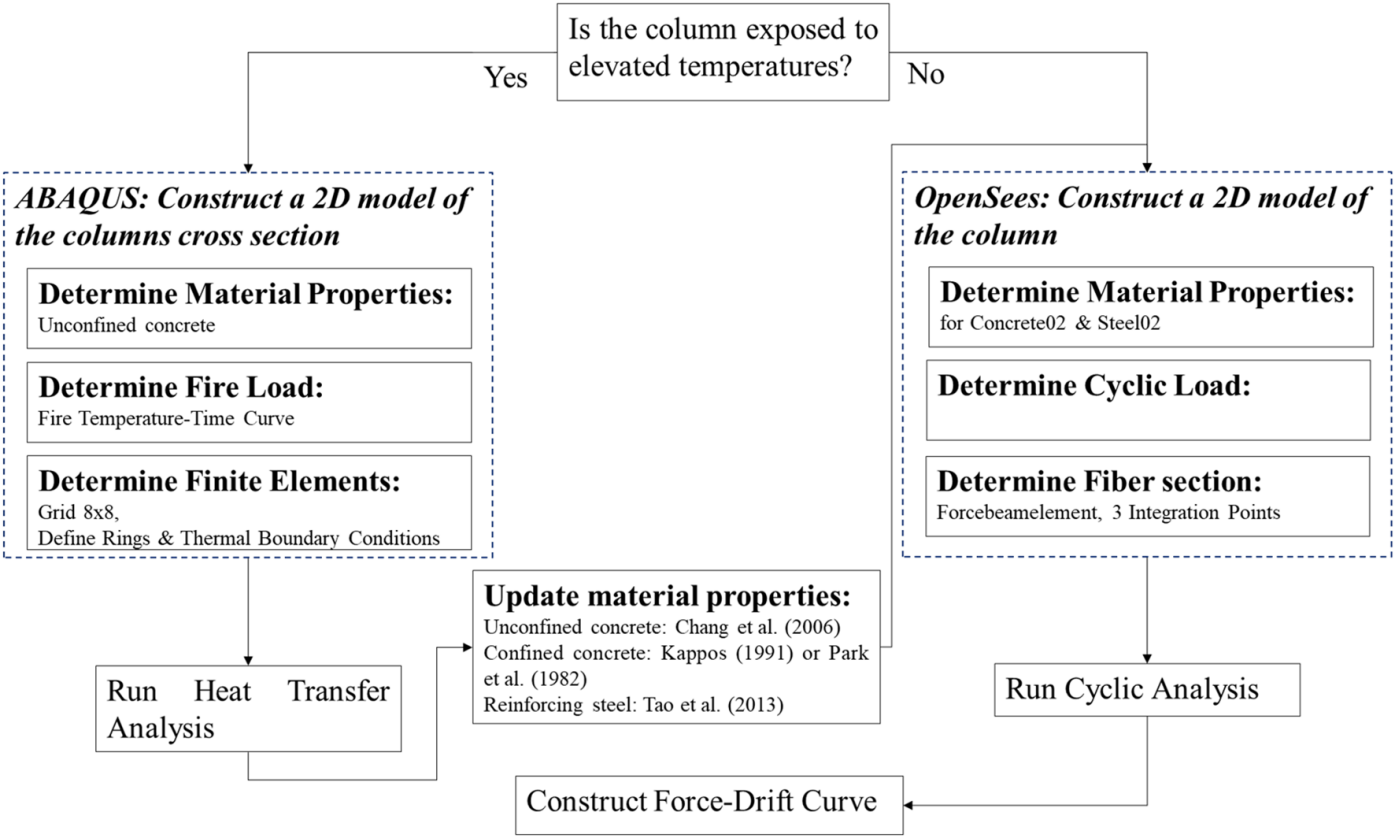
The framework proposed in this study in order to model the post-fire seismic behaviour of a non-seismically designed RC column.

The framework for modelling the seismic response of a non-seismically designed RC column that has previously been exposed to fire is presented in Fig. 1. Although similar frameworks have been proposed in the literature, the one proposed in this study focuses in greater detail on how to update the material properties. According to the proposed framework, a heat transfer analysis on the column is first conducted to estimate the temperature penetration in the column exposed to a given temperature–time curve. The modelled peak temperature of the whole heating–cooling phase is used to determine the deterioration of the steel and concrete properties based on existing material models. The seismic behaviour of the column, described by the residual post-fire material properties, is then investigated. Different material models are investigated in the following sections, and recommendations are made on which models result in the best approximation of the force-drift response observed in experiments of RC columns that have been tested under sequential fire and cyclic loading. It should be mentioned that the framework also allows for the case where the column is exposed only to cyclic loading without having been exposed to elevated temperatures.

In what follows, the models necessary for the thermal and seismic analysis are described and special focus is given to the modelling of the material properties.

### Heat transfer analysis

**Figure 2:**
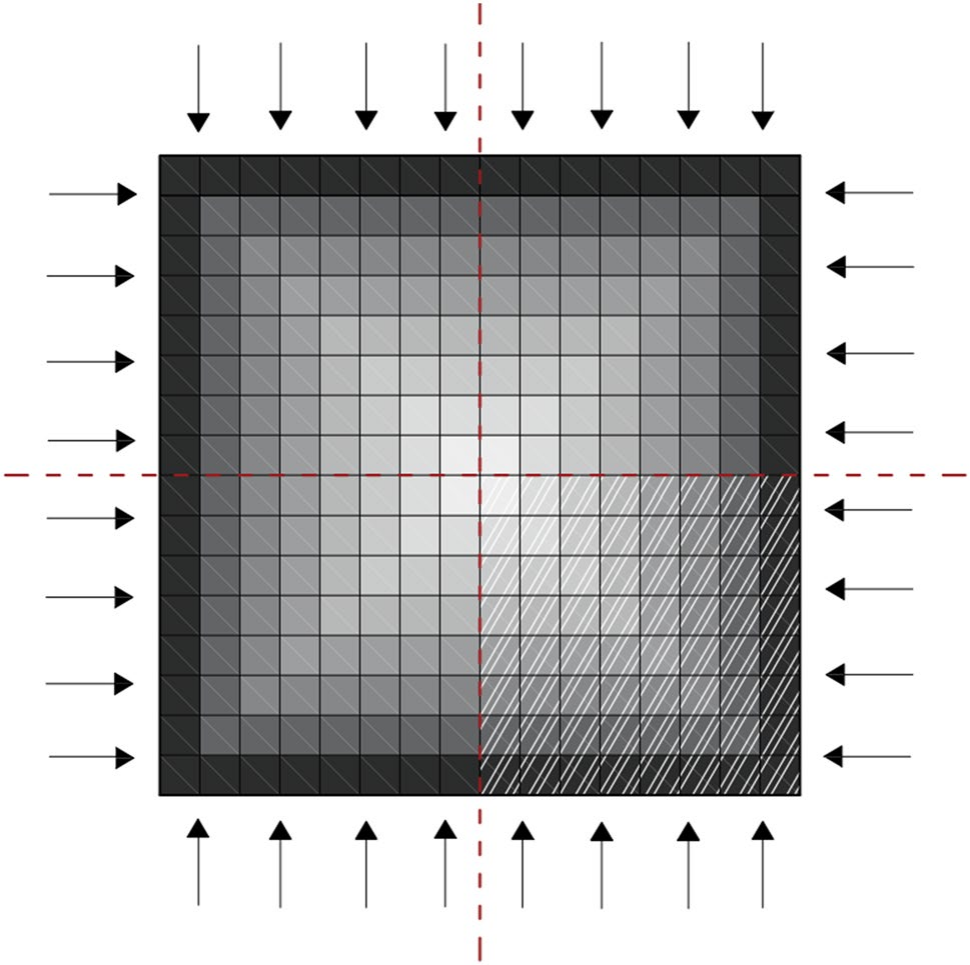
Example of 2D model of column’s square cross section, used for the thermal analysis.

A transient heat transfer analysis is performed to determine the heat penetration across the column for the duration of a specified fire scenario, expressed in terms of a fire temperature–time curve. Despite the availability of finite element software (e.g., SAFIR) which specialise on fire analysis, the heat transfer analysis is performed here in ABAQUS, a generic finite element software widely used by the engineering community. The analysis is based on the simplified consideration, in line with the experimental procedure, that the column is uniformly exposed to fire, with all four sides being subjected to elevated temperatures along its height. This allows for a simple 2D model of the column’s cross-section to be used to predict the temperature penetration across the column. By further considering that the cross-section is square and uniformly exposed to fire, in this case, symmetry can be exploited and only a quarter of the cross-section needs to be modelled. In general, the size of the mesh depends on the complexity of the structure. Nonetheless, due to the simplified model used here and the aggregation of the results (see "Post-fire residual materials properties" section for more details), a relative coarse mesh, e.g. an 8 × 8 grid as depicted in Fig. 2, can be used. Concrete is a highly insulating material and this means that when exposed to fire, its temperature is raised at a slower rate than the reinforcing steel. For this reason, only the concrete is modelled ignoring the reinforcing steel, whose maximum temperature is assumed equal to the closest concrete layer, in line with existing literature (e.g.^11^).

The key output from the heat transfer analysis is the maximum temperature experienced by each element throughout the heating–cooling phase. The surface of the concrete will experience higher temperatures than its core. Moreover, the corners of the square section will experience higher temperature than the middle. This is taken into account in estimating the temperature experienced by the longitudinal bars, which are assigned the maximum temperature of their closest element. By contrast, the properties of the concrete and transverse reinforcement are based on a procedure similar to the one proposed in^12^ for the estimation of the axial load capacity of a composite column. According to this approach, the cross-section is divided into rings of equal thickness, where the temperature is considered uniform. The maximum temperature for each ring is then determined by averaging the maximum temperature experienced by each element within the examined ring. The aforementioned values of maximum temperature will determine the level of degradation of the reinforcing steel and concrete properties, as described in "Post-fire residual materials properties" section.

### Seismic analysis

The nonlinear seismic behaviour of the column is modelled in OpenSees by a simplified 2D model, which is constructed with a single force-based fibre beam column element. This type of element considers that the nonlinearities are distributed across the length of the column in the form of fibre cross-sections at a user-specified number of integration points. Each fibre cross-section is assembled by assigning uniaxial stress–strain relationships to the concrete of the cover and core, as well as to the longitudinal reinforcement bars. The stress–strain relationship of the concrete in the core accounts for the confinement effect of the transverse reinforcement.

Stress–strain models frequently adopted in the construction of analytical fragility curves for RC structures to express the behaviour of the materials in cyclic loading such as Steel02 (e.g.^13,14^) and Concrete02 (e.g.^13–15^) are also adopted here. The Steel02 is a bi-linear response envelope based on the Giuffré–Menegotto–Pinto^16^ model, which accounts for the strain hardening and has three key properties: the yield strength, the modulus of elasticity and the strain hardening ratio. The Concrete02^17^ model simulates the tensile strength of the concrete. Key properties for the selected concrete model are the maximum compressive strength and corresponding strain, the ultimate strength and corresponding strain, and the tensile strength. Concrete02 is used to express the nonlinear behaviour of both the unconfined concrete in the cover as well as the confined concrete in the core. In line with existing literature (e.g.,^1,9^), the values of the aforementioned key variables for both steel and concrete are estimated either by experimental data, or where this is not possible, through existing stress–strain models (details in "Post-fire residual materials properties" section). With regard to the confined concrete, key properties are estimated using existing confined concrete stress–strain models for monotonic loading.

Having constructed the model, the seismic performance of the column exposed to cyclic loading and a constant axial load is modelled with the aim of determining the drift-force relationship of the column.

### Post-fire residual materials properties

The procedure used to estimate the values of the key properties for the steel and concrete models in OpenSees, assuming that the column was exposed to elevated temperatures and allowed to cool down, is presented in this section.

#### Reinforcing steel

The post-fire residual material properties of the longitudinal and transverse reinforcing steel are estimated by the Tao et al.^18^ model based on the maximum temperatures assigned to the longitudinal and transverse reinforcement from the fire analysis described in "Heat transfer analysis" section. According to this model, the steel yield strength and the ultimate stress reduces after being exposed to temperatures of 500 °C and above.

#### Concrete

The post-fire residual properties of the concrete in the cover and core are determined from the Chang et al.^19^ model, using as input the maximum temperature for each ring in the cross-section.

The overall residual maximum compressive strength of the unconfined concrete in the cover,$$ f_{{c,T_{Cover} }}$$, for a given fire scenario is estimated as the weighted average of the strength determined for each ring in the cover, as:

1 $$ f_{{c,T_{Cover} }} = \mathop \sum \limits_{i = 1}^{n} \left( {A_{i} f_{{c,T_{i} }} } \right)/A_{cover} $$

where $f_{{c,T_{i} }}$ is the peak residual compressive strength for cross-section ring *i*; $A_{i}$ is the area of ring *i*; $A_{cover}$ is the area of the cover. Similarly, the cover’s strain corresponding to the maximum compressive strength, $\varepsilon_{{c,T_{Cover} }}$, is also estimated as the weighted average of the strain for each ring in the cover as:

2 $$ \varepsilon_{{c,T_{Cover} }} = \mathop \sum \limits_{i = 1}^{n} \left( {A_{i} \varepsilon_{{c,T_{i} }} } \right)/A_{cover} $$

where $\varepsilon_{{c,T_{i} }}$ is the strain corresponding to the peak residual compressive strength for ring *i*; The stress–strain relationship proposed by Chang et al.^19^ is then used to obtain the straincorresponding to the ultimate compressive strength, $\varepsilon_{{cu,T_{Cover} }}$, which is considered equal to 10% of its maximum value (i.e., $f_{{cu,T_{Cover} }} = 0.10f_{{c,T_{Cover} }}$). Finally, the tensile strength of the unconfined concrete is reduced according to the increase in the temperature using the equations in Chang et al.^19^. The overall reduction of the tensile strength for the concrete in the cover is considered equal to:

3 $$ f_{{t,T_{Cover} }} /f_{{t,20^{o} C_{Cover} }} = \mathop \sum \limits_{i = 1}^{n} \left( {A_{i} f_{{t,T_{i} }} /f_{{t,20^{o} C_{Cover} }} } \right)/A_{cover} $$

With regard to the core, the determination of the post-fire residual properties (i.e., $f_{{c,T_{Core} }}$,$\varepsilon_{{c,T_{Core} }}$, $f_{{t,T_{Core} }}$, $f_{{cu,T_{Core} }}$,$\varepsilon_{{cu,T_{Core} }}$) is hindered by the lack of appropriate confined concrete models. To overcome this problem, a simplified approach is proposed for use, which is in line with available literature^1,9^. Within the proposed simplified approach, the Chang et al.^19^ model is used to estimate the residual properties of the unconfined concrete for each ring of the core. Similar to the procedure used for the cover, Eqs. (1)–(3) are adopted to estimate the residual properties of the concrete in the core assuming it is unconfined. These residual properties are then used as input to a chosen existing confinement model to determine the confined concrete properties of the core. Given the lack of confined concrete models for elevated temperatures, it is a key assumption in this study that confined material models validated by experimental results on ambient temperatures can be used to estimate the material properties of confined concrete previously exposed to elevated temperatures and which have degraded properties at ambient temperature. There are numerous confinement models available for concrete in the literature, which are typically validated with experimental data at ambient temperatures. A few of the most widely used in the field of earthquake engineering are adopted in a sensitivity study to understand the influence on the post-fire cyclic response of columns modelled with different confinement models. The results of the sensitivity study show that either the Kappos^20^ or the Park et al.^21^ models can be used to determine the properties of the confined concrete in the core in the case of fire-affected columns subjected to cyclic loading.

## Validation of the proposed simplified model

The reliability of the model, developed in "Framework for modelling post-fire cyclic behaviour of RC column" section, is validated through its application to a pre-code RC column and comparison of the results with experimental counterparts.

### Description of specimens

**Figure 3:**
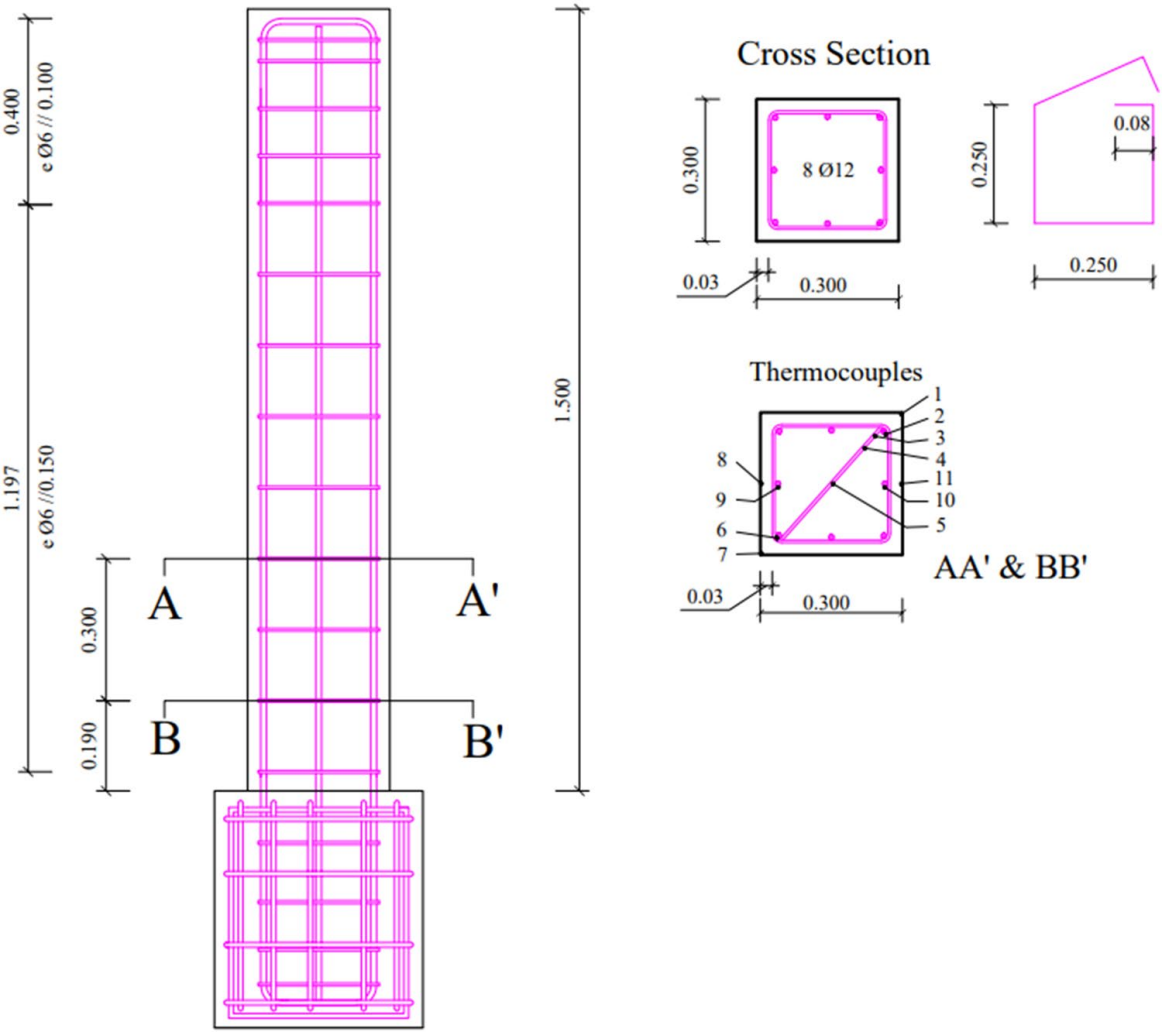
Details of specimens.

Three full-scale square RC columns are constructed with the geometrical properties and reinforcing details depicted in Fig. 3. The specimens are cantilevers with 1.5 m height and cross-section dimensions 0.30 m × 0.30 m. Each column is connected to a square RC foundation block of cross-section dimensions 0.44 m × 0.44 m and height 0.5 m. The columns are constructed according to the pre-1970s Portuguese code^10^. The clear cover is 0.025 m, and the longitudinal reinforcement comprises 8 ribbed bars of 12 mm diameter and quality A400. Transverse reinforcement of 6 mm diameter and with 90° hooks is also present in each column (see Fig. 3). The centre-to-centre spacing of the transverse reinforcement is 0.15 m in the lower 0.40 m of the column, and 0.10 m in the rest of the column. The material properties of the unconfined concrete and reinforcing steel, depicted in Table 1, are obtained experimentally for ambient temperature. It should be mentioned that calcareous aggregates have been used for the concrete and its moisture content in volume is Gravel/Sand/Cement is 3.3/3/1, respectively. Tests performed on concrete samples after 10 months of their casting showed that the mean compressive strength of concrete is equal to 33.5 MPa.

**Table 1:** Experimental mechanical properties of longitudinal and transverse reinforcement.

Scenario	Type	*f*_*sy*_ (MPa)	*ε*_*sy*_ (%)	*E*_*s*_ (MPa)	*f*_*su*_ (MPa)	*ε*_*su*_ (%)	*b*_*s*_
C	Longitudinal	445	0.23	195,000	571	17.5	0.0035
	Transverse	540			639	18.0	

*f*_*sy*_: yield strength of steel.

*ε*_*sy*_: yield strain of steel.

*E*_*s*_: modulus of elasticity of steel.

*f*_*su*_: ultimate strength of steel.

*ε*_*su*_: ultimate strain of steel.

*b*_*s*_*:* strain hardening of steel.

**Figure 4:**
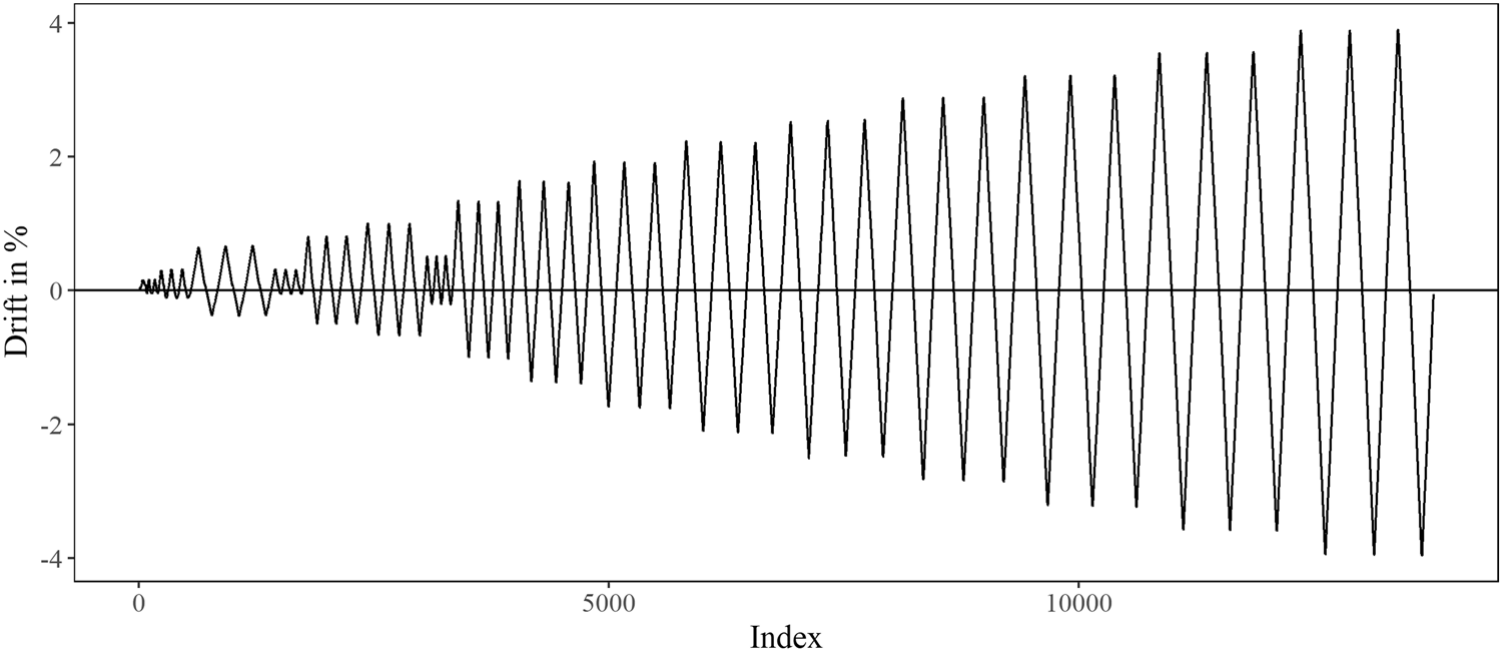
Loading protocol.

**Figure 5:**
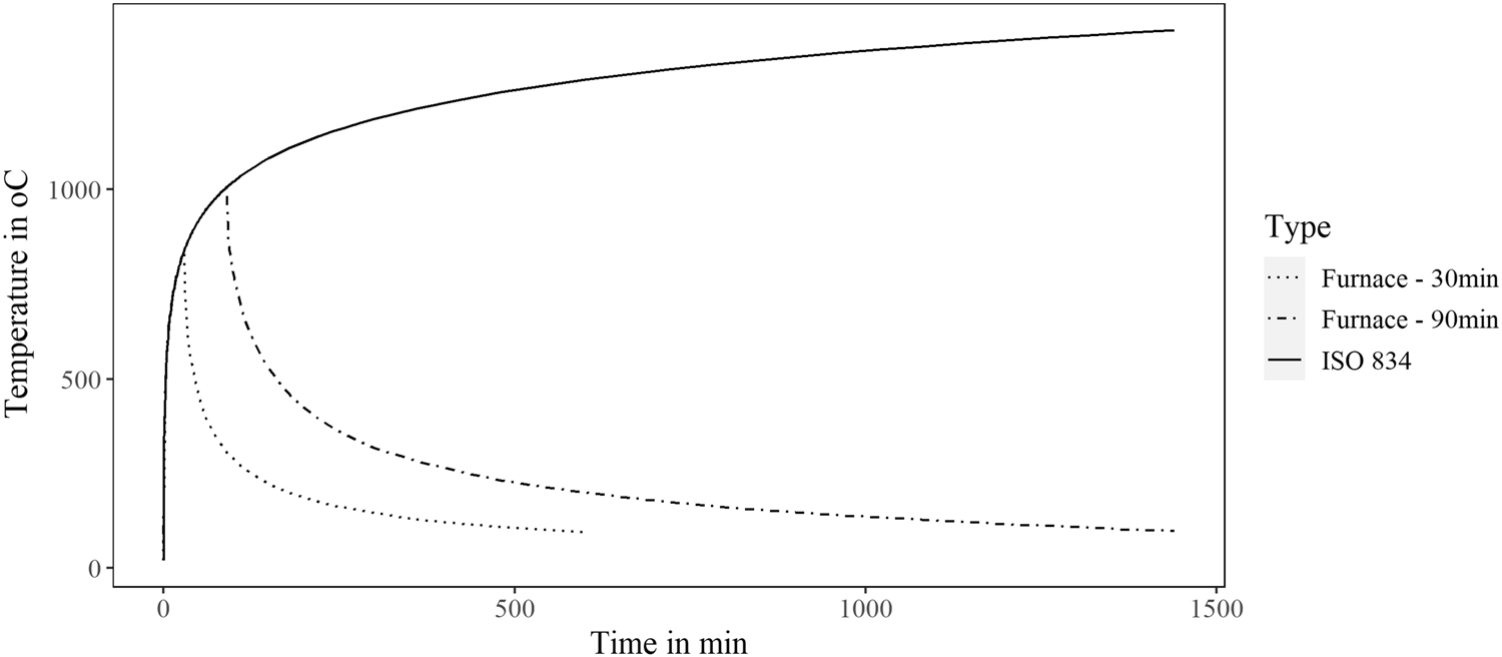
Experimental fire-temperature curves.

**Figure 6:**
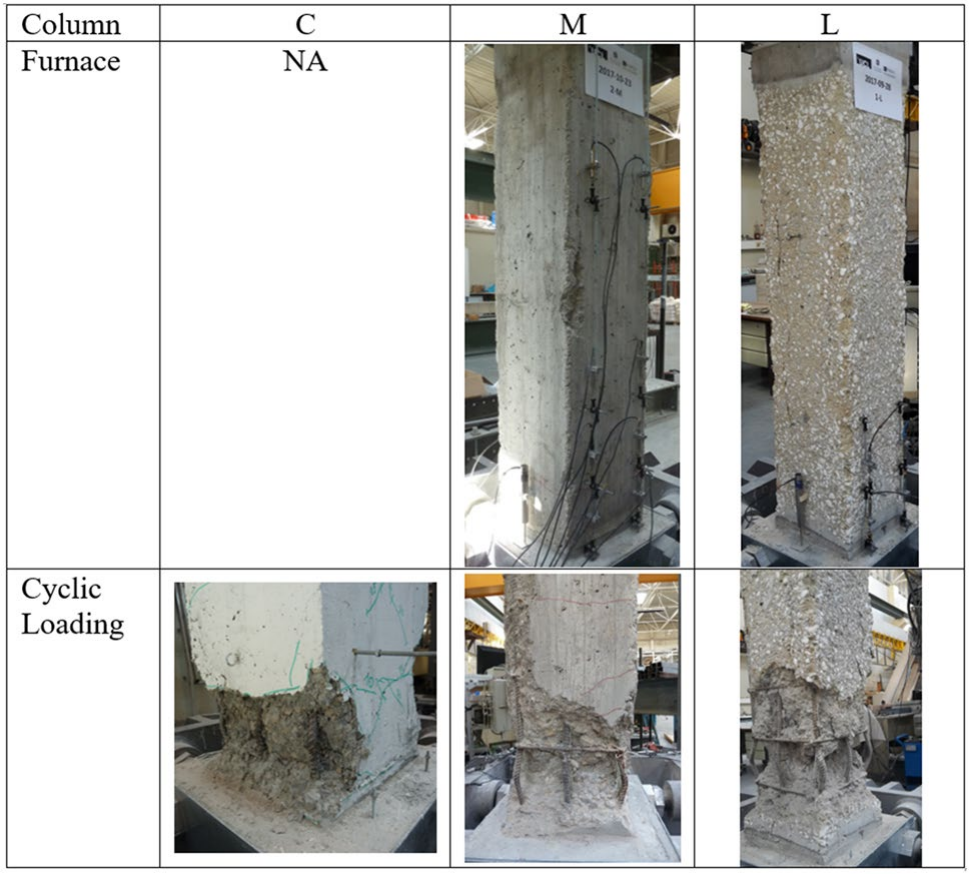
The three tested specimens (i.e., C, M and L) after being exposed to elevated temperatures (where applicable) and after their failure to cyclic loading.

In the experimental programme, the specimens were all cast at the same time and cured for at least 6 months at ambient laboratory temperature and relative humidity conditions before the fire exposure. The first specimen (reference specimen termed ‘C’ in this study) is subjected to uniaxial cyclic loading (see Fig. 4 for the loading protocol) under a constant axial load equal to 410 kN approximately 6 months after it has been cast. The remaining two columns (termed ‘M’ and ‘L’ herein) are first exposed to ISO-834 time–temperature curves (as depicted in Fig. 5) in a furnace of 30 min and 90 min duration without any applied load (or restraint). The two columns are allowed to cool down in the furnace to ambient temperature, and then tested after approximately 4 months under the same uniaxial cyclic loading (see Fig. 4) and axial load as the control specimen. All columns are tested to failure. Figure 6 depicts the state of the columns at the end of their test in the furnace. It can be noted that after being exposed to 30 min fire curve the unconfined concrete in the cover appears to have suffered hairline cracks. By contrast, exposure to the 90 min fire curve results in clear signs of spalling (ranging between 10–20 mm) distributed along the length of the column and on each column face, with cracks on the cover seen on one face of the column. Further information on the concrete cover damage sustained is provided in^2^. During the uniaxial cyclic loading, all three columns failed with the formation of a plastic hinge at the base of the column, also depicted in Fig. 6. The 90 min fire exposure affected the concrete properties to a much greater extent than the 30 min fire. Consequently, the steel yield stress, and compressive and tensile strengths of the concrete in the 90 min fire were lower. These reduced properties and greater yield penetration resulted in longer plastic hinges.

**Figure 7:**
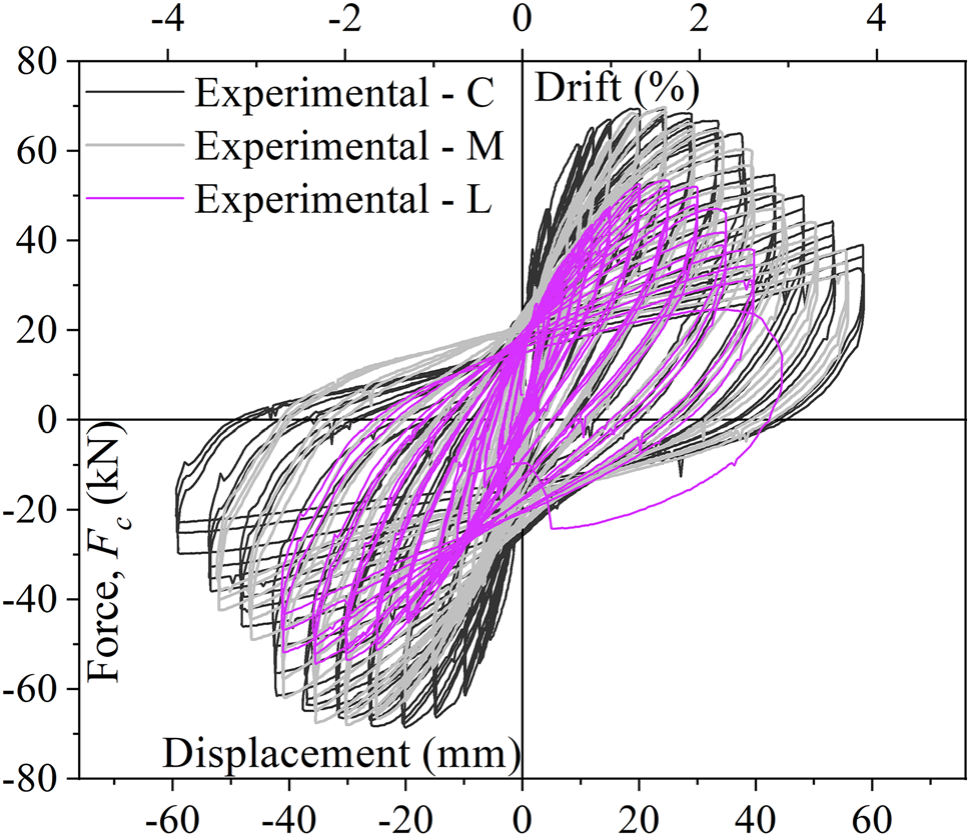
Lateral load–displacement relationship curves based on the performance of the three tested specimens (i.e., C, M and L).

The experimental hysteretic curves for column C, M and L are compared in Fig. 7. It can be seen, that the 30 min exposure of the column to fire results in lower initial stiffness for column M compared to the reference column C. By contrast, the peak force reached during the cyclic test, as well as the post-peak degradation, remain approximately the same for both columns. When the exposure to fire is increased to 90 min, it can be noted that apart from having a lower initial stiffness, the peak force and the ultimate displacement are also significantly lower for column L than for C. By contrast, the degree of degradation remains similar for all three columns. Α more detailed discussion on the behaviour of the three columns during the experiments can be found in^2^. This behaviour the proposed model aims to capture by applying the proposed framework in what follows.

### Heat transfer analysis

**Figure 8:**
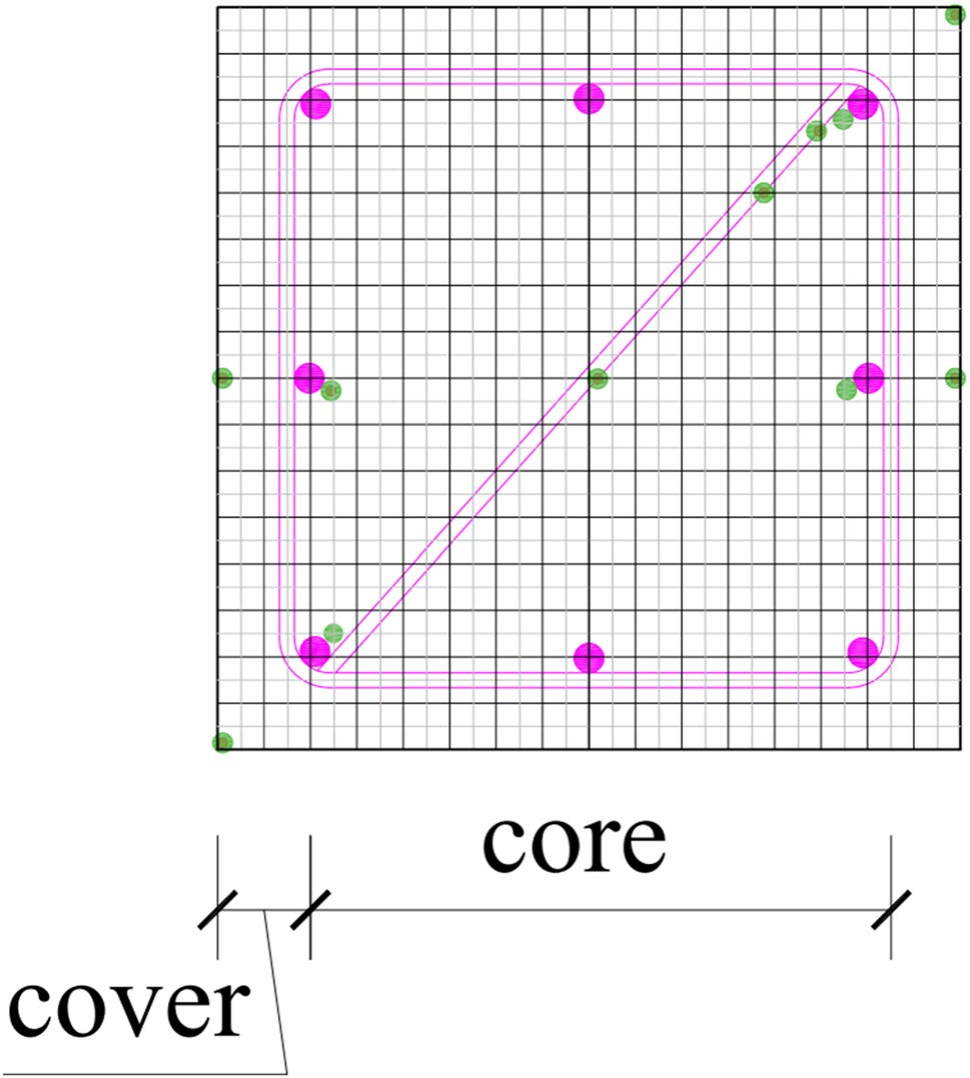
2D concrete model used for the thermal analysis (the transverse and longitudinal reinforcement as well as the location of the thermocouples are included for guidance).

A transient heat transfer analysis of the column is performed with ABAQUS using a simplified model as outlined in "Heat transfer analysis" section for the two fire scenarios (i.e., fire duration 30 min and 90 min), determined in "Description of specimens" section. A quarter of the cross-section is modelled in 2D and subdivided in 8 × 8 elements as depicted in Fig. 8. The thermal properties of the concrete necessary for this analysis are its density, specific heat capacity and its conductivity. Their values are assumed to vary with temperature and the curves are obtained from the recommendations of Eurocode 2^22^. For ambient temperature, the values considered are: *ρ* = 2,300 kg/m^3^, c = 900 J/kgK and *λ* = 1.9 W/mK, respectively. The emissivity level of concrete is equal to 0.7 and the convective flux coefficient is considered equal to 25 W/m^2^K. To allow the direct comparison of experimental and analytical results, the furnace fire temperature–time curves depicted in Fig. 5 have been used.

The validity of the assumptions made in the modelling can be tested by comparing the temperature recorded by the thermocouples during the two experiments M and L to their counterparts recorded by the mesh element closest to their exact location. This, however, requires a finer mesh than the 8 × 8 adopted here. The authors have conducted this comparison in^23^ using a finer 16 × 16 mesh and the results were found to be satisfactory.

Having modelled the temperature distribution to the cross-section of the columns M and L, the maximum temperature experienced by the longitudinal reinforcement is determined by the mesh element closest to each bar. The maximum temperatures experienced by the concrete in the cover and core as well as the transverse reinforcement, required to update the material properties as described in "Post-fire residual materials properties" section, are determined as follows. The column’s cross-section is divided into 16 rings, which are equal in width and the temperature is considered uniform across each ring. For a given ring, the maximum temperature of the heating–cooling cycle is determined as the average of the maximum temperature of each element. A summary of the maximum temperature at each ring in the cover and core are depicted in Table 2.

**Table 2:** Maximum temperature experienced by the element closest to the longitudinal bars as well as each ring in the cover and core.

Column	Cover		Core					
	Ring_1_ (outer)	Ring_2_	Ring_3_	Ring_4_	Ring_5_	Ring_6_	Ring_7_	Ring_8_ (centre)
M	539.1	289.5	208.5	174.3	160.3	156.9	156.2	156.1
L	836.4	576.5	436.6	376.1	354.8	349.3	347.9	347.7

### Material properties

#### Reinforcing steel

For ambient temperatures, the properties of the reinforcing steel are based on the experimental data, depicted in Table 1. For the two post-fire scenarios, however, the residual properties of the reinforcing steel are determined by the model of Tao et al.^18^ and the values are depicted in Table 3. It should be noted that the location of the longitudinal reinforcement (i.e., whether it is in the corner or the middle of the cross section) affects the reinforcing bar properties only for the most extreme scenario considered, and even in this case the changes are small (i.e., maximum difference in properties 13%). The post-fire residual properties of the transverse reinforcement are also estimated by the Tao et al.^18^ model and are presented in Table 3.

**Table 3:** Key properties of reinforcing steel for the two post-fire scenarios.

Column	Type	Location	maxT (°C)	*f*_*sy*_ (MPa)	*E*_*s*_ (MPa)	*f*_*su*_ (MPa)	*ε*_*su*_	*b*_*s*_
M	Longitudinal	Corner	415.5	445	195,000	571	0.18	0.35%
		Middle	252.7					
	Transverse	–	289.5	540				
L	Longitudinal	Corner	754.1	386	188,558	501	0.16	0.38%
		Middle	510.8	443	194,726	568	0.18	0.36%
	Transverse	–	576.5	519				

#### Concrete

For column C, the maximum compressive strength of the unconfined concrete is experimentally determined to be equal to 33.5 MPa and the corresponding strain is determined by the provisions of Eurocode 2^22^. Eurocode 2^22^ is also used to determine the tensile strength of the unconfined concrete. The ultimate strength and strain of the unconfined concrete are determined by the Chang et al.^19^ stress–strain model. With regard to the confined concrete, the values of key properties are estimated using four confined concrete stress–strain models: the Park, Priestley and Gill^21^, Kappos et al.^20^, Mander et al.^24^ and Chang and Mander^25^. These four models are widely used in the field of earthquake engineering to determine the properties of the confined concrete in the core of reinforced concrete columns subjected to cyclic loading. Furthermore, the latter two confinement models have been used by the past two studies that have modelled the impact of sequential fire and earthquakes on the performance of vertical structural elements^1,8^. This is done to determine which concrete confinement model can be used to best represent the post-fire behaviour of the concrete. Furthermore, the assumption that the transverse reinforcement offers no confinement is also explored. In the latter case, the Chang et al.^19^ stress–strain model is additionally used to determine the core concrete properties. The values of the key properties of the concrete for both the core and cover concrete for modelling the cyclic performance of the column C are depicted in Table 4.

**Table 4:** Key properties of concrete for the three scenarios.

Scenario	Element	Type	Model	*f*_*cy*_ (MPa)	*ε*_*cy*_ (%)	*f*_*cu*_ (MPa)	*ε*_*cu*_ (%)	*factorf*_*t*_
C	Cover	Unconfined	Chang et al.^19^	33.5	0.21	3.4	0.72	1.0
	Core	Unconfined	Chang et al.^19^	33.5	0.21	3.4	0.72	
		Confined	Park et al.^21^	35.1	0.22	7.0	0.86	
			Kappos et al.^20^	35.4	0.24	8.9	0.83	
			Mander et al.^24^	35.5	0.27	7.1	3.77	
			Chang and Mander^25^	35.5	0.27	4.0	0.82	
M	Cover	Unconfined	Chang et al.^19^	21.4	0.41	2.1	0.76	0.6
	Core	Unconfined	Chang et al.^19^	30.0	0.21	3.0	0.57	0.8
		Confined	Park et al.^21^	31.6	0.22	6.3	0.89	
			Kappos et al.^20^	31.8	0.24	8.0	0.86	
			Mander et al.^24^	32.1	0.28	6.4	5.71	
			Chang and Mander^25^	32.0	0.28	6.2	0.84	
L	Cover	Unconfined	Chang et al.^19^	15.2	0.64	1.5	0.90	0.2
	Core	Unconfined	Chang et al.^19^	23.2	0.31	2.3	0.66	0.6
		Confined	Park et al.^21^	24.8	0.33	5.0	0.92	
			Kappos et al.^20^	24.9	0.35	6.2	0.91	
			Mander et al.^24^	25.3	0.45	17.7	2.69	
			Chang and Mander^25^	25.1	0.43	10.9	1.30	

With regard to the post-fire scenarios, the residual properties of unconfined and confined concrete are determined by the procedure outlined in "Concrete" section and a summary of the estimates is presented in Table 4. For model ‘L’, spalling of between 10-20 mm was noted following the fire exposure in the experiments. Accordingly, the material properties in Table 4 are estimated assuming half the original concrete cover width (i.e., 0.0125 m of cover rather than 0.025 m).

**Figure 9:**
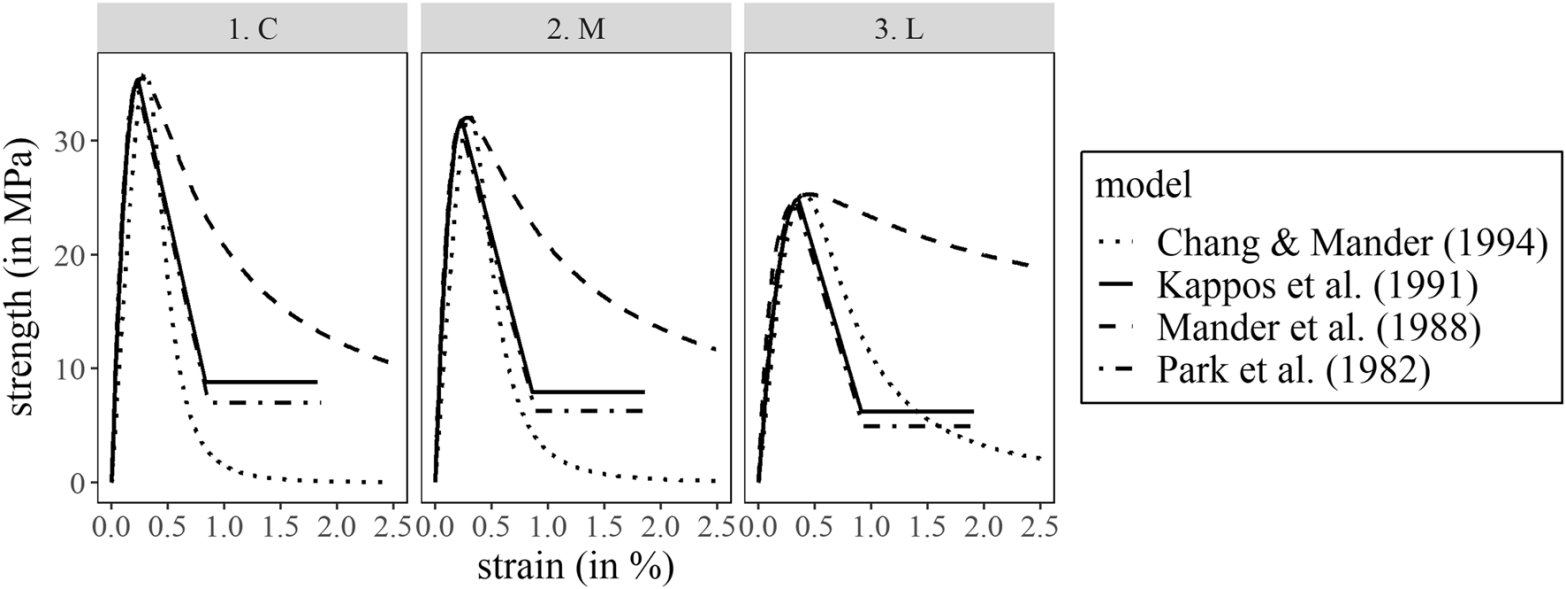
Comparison of experimental vs modelled maximum temperature for the two fire scenarios (i.e., affecting columns M and L) at the location of the 11 thermocouples.

The differences between the four concrete confinement models are depicted in Fig. 9 by plotting their strength-strain relationships. Park et al.^21^ proposed a piece-wise function, which describes the ascending branch as a parabola and the descending branch as a straight line and plateaus at a given threshold (i.e., 20% of the maximum strength). This simplified analytical model has been widely used to describe the confinement as it has been based on a sufficient number of reliable experiments. The relationship proposed by Kappos^20^ is a variation of the one proposed by Park et al.^21^. By contrast, Mander et al.^24^ and Chang and Mander^25^ proposed a single relationship which described both the ascending and descending branch to estimate the compressive strength of the confined concrete subjected to uniaxial concentric loading until the first fracture of the transverse reinforcement. In Fig. 9, the maximum compressive strength and its corresponding strain is similar for all three models. However, the strength degradation is much sharper for the Park et al.^21^ and Kappos et al.^20^ models and less so for the Mander et al.^24^ model for all three scenarios. By contrast, the strength degradation is sharper for Chang and Mander^25^ model for scenarios C and M when compared to the other three models. However, it is less steep than Kappos^20^ and Park et al.^21^ for column L. The impact of these differences in the cyclic behaviour of the columns is examined in "Results" section.

### Seismic loading

The nonlinear seismic behaviour of the three columns is modelled in OpenSees by following the modelling assumptions outlined in "Seismic analysis" section. A generic force-based fibre beam column element is used to model the cyclic performance of each column. The Steel02 and Concrete02 models are used to represent the behaviour of reinforcing bars and concrete in the cover and core based on the values in Tables 3 and 4 for the C, M and L columns. The results were found to be sensitive to the number of integration points. In this study, three integration points are used for each analysis as this number is found to produce results which best match the experimental results.

### Results

Having simulated the seismic behaviour of the three columns (i.e., C, M and L) using five different models for the concrete properties in the core, the numerical results are compared to their experimental counterparts. This comparison is used to understand how well the models predicted the behaviour of the columns and to identify the model that fits the experimental data best. The comparisons are based on features of the hysteretic curve as well as on the dissipated energy evolution.

#### Hysteretic curves

The differences between the experimental and numerical results are examined here by comparing the lateral force-drift relationship of the columns under cyclic loading.

**Figure 10:**
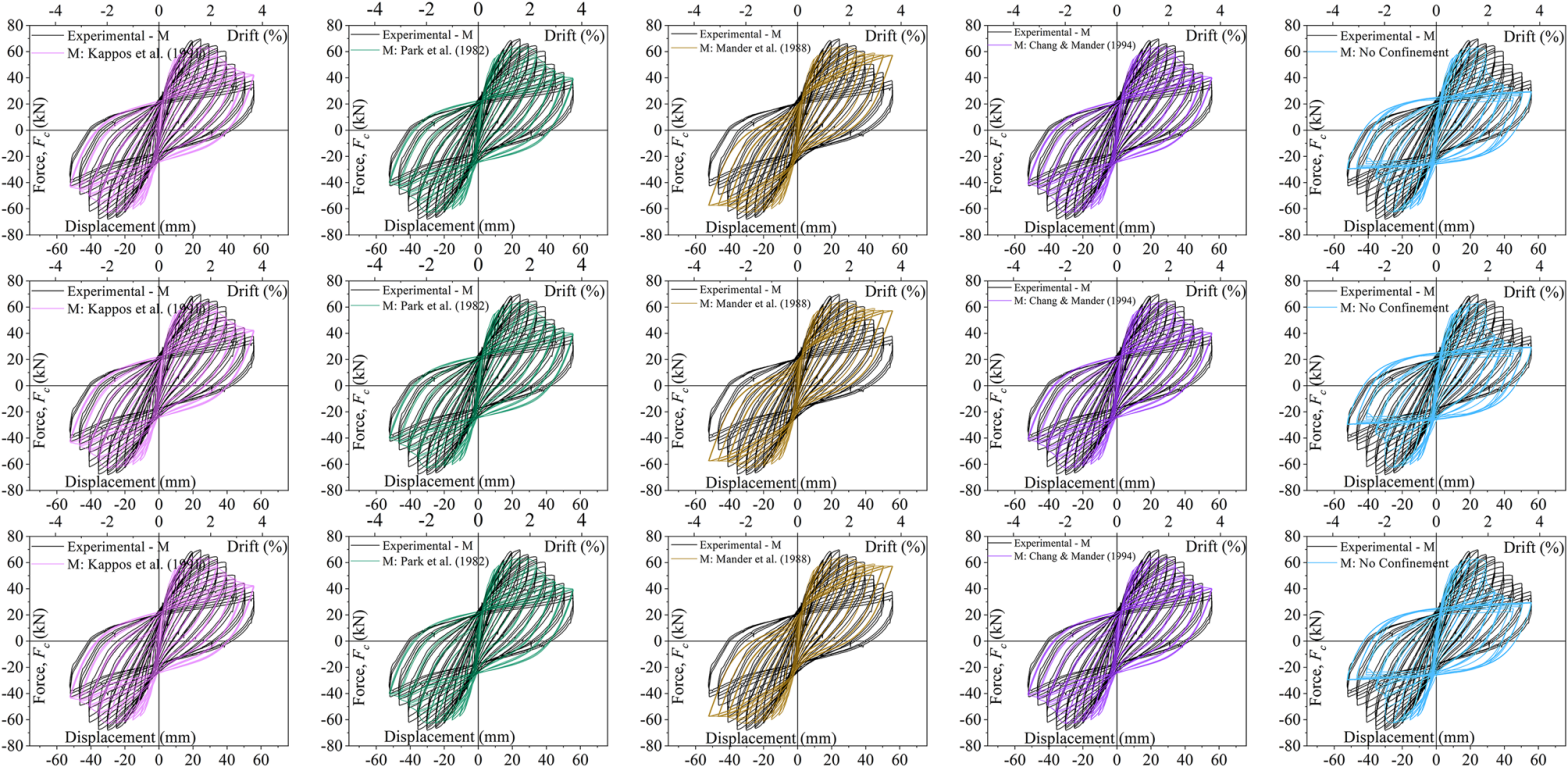
Lateral load–displacement relationship: column C (top row); column M (middle row); column L (bottom row).

**Figure 11:**
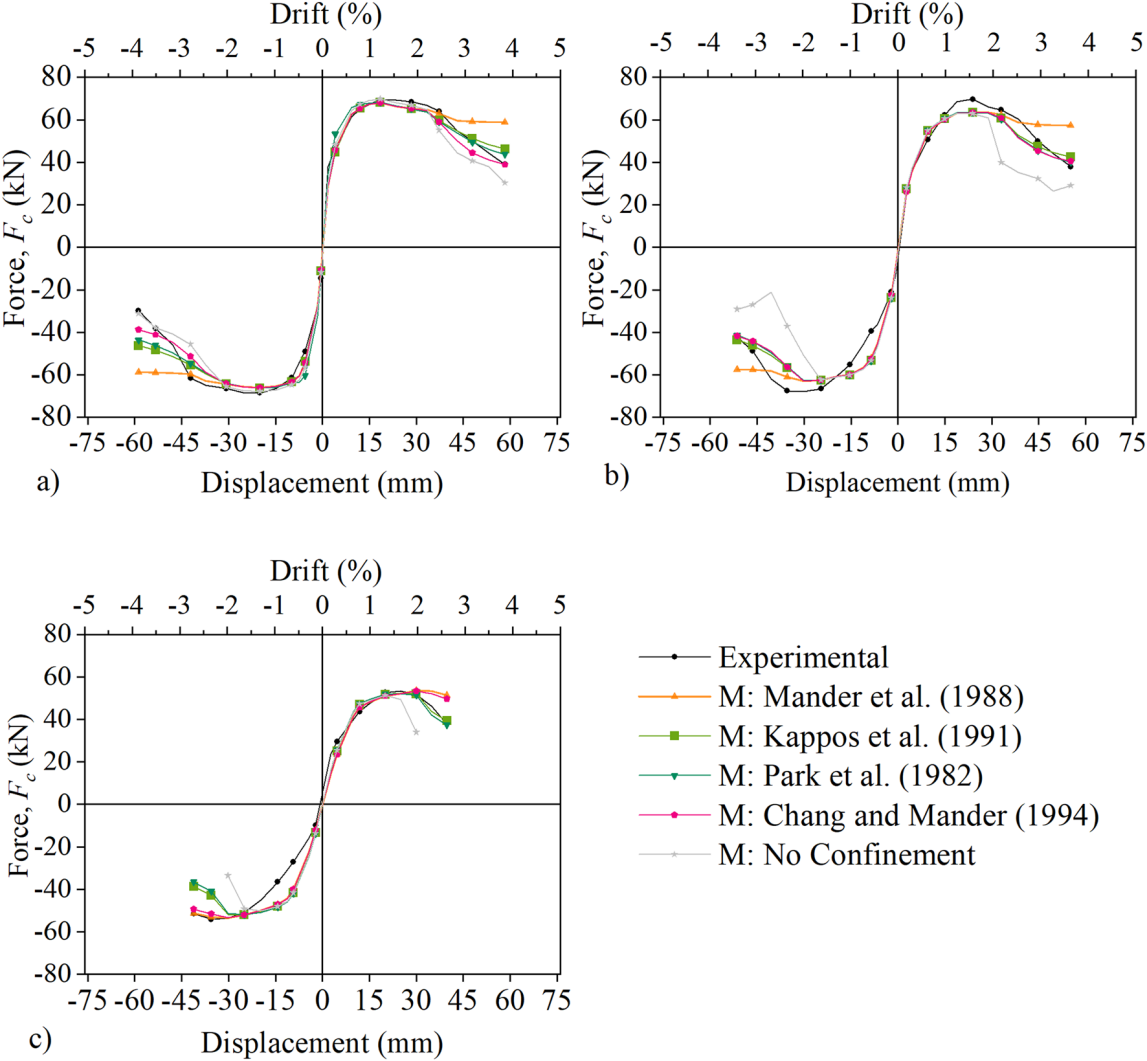
Lateral Force vs Displacement envelops: (**a**) column C, (**b**) column M, (**c**) column L.

In Fig. 10, the lateral force–displacement curves are also used to assess how close the five numerical models are to the experimental results for the three columns: C, M and L. It can be noted that all models behave approximately the same until the peak lateral force is reached, after which significant differences are notable. This can also be clearly seen by the comparison of the experimental and numerical lateral force–displacement envelops in Fig. 11a–c. The Park et al.^21^ as well as the Kappos et al.^20^ models appear to capture well the post-peak behaviour of all three columns as depicted in Fig. 11a–c. Instead, the Chang and Mander^25^ model captures well the post-peak behaviour for columns C and M but fails to capture the strength degradation for L. The Mander et al.^24^ model also fails to represent the strength degradation past the peak lateral force for all three columns. Finally, the model which assumes no confinement systematically under-predicts the experimental behaviour of all three columns past the peak force. This highlights the important role of (limited) confinement in the post-peak behaviour of the pre-code columns, even when the transverse reinforcement has 90° hooks.

In Table 5, a summary of key values (for the positive direction of the columns’ drift) of the cyclic test is shown for the three columns. The summary includes the peak lateral force (*F*_*c,max*_), and its corresponding drift (*d*_*c,max*_), the ultimate lateral force (*F*_*c,ult*_), and corresponding drift (*d*_*c,ult*_), the drift at the yield point (*d*_*c,y*_), and the displacement ductility at ultimate point (*μ*_*Δ,ult*_). The ultimate point is considered here as the point at which the strength drops by 20% of *F*_*c,max*_, according to^26^. Due to this assumption, the ultimate force and drift for the Mander et al.^24^ model for all three columns and for the Chang and Mander^25^ for column L cannot be estimated, as these models do not predict strength degradation. With regard to *d*_*c,y*_, the yield displacement is calculated according to Annex B.3 of EC8-1^27^. For each column, an elastic-perfectly plastic relationship is fitted to the experimental or numerical lateral load–displacement envelope up to the ultimate point, ensuring the following requirements are satisfied: (1) the areas under and above the envelope curve must have the same values; and (2) the area under (or above) the envelope curve is the lowest possible^28^. Finally, the displacement ductility at ultimate point is equal to the ratio of *d*_*c,ult*_ over *d*_*c,y*_.

**Table 5:** Maximum and ultimate lateral force and corresponding drift, drift at yield strength and ductility at ultimate strength.

Source	Column	*F*_*c,max*_	*d*_*c,max*_	*F*_*c,ult*_	*d*_*c,ult*_	*d*_*c*_*,*_*y*_	*μ*_*Δ,ult*_	Diss. energy
		(kN)	(%)	(kN)	(%)	(%)		(kNm)
Experim	**C**	**69.4**	**1.2**	**55.5**	**2.8**	**0.4**	**7.4**	**53.7**
Anal	Mander et al.^24^	68.1	1.2	–	–	0.4	**–**	**–**
	Kappos et al.^20^	68.3	1.2	54.6	2.9	0.4	7.8	42.4
	Park et al.^21^	68.3	1.2	54.6	2.8	0.4	7.6	39.7
	Chang and Mander^25^	68.1	1.2	54.5	2.7	0.4	7.3	35.5
	No confinement	68.3	1.2	54.6	2.4	0.4	6.5	27.3
Experim	**M**	**69.7**	**1.6**	**55.8**	**2.7**	**0.7**	**4.2**	**45.1**
Anal	Mander et al.^24^	64.3	1.2	–	–	0.6	**–**	**–**
	Kappos et al.^20^	64.4	1.2	51.5	2.7	0.6	4.2	33.9
	Park et al.^21^	64.5	1.2	51.6	2.6	0.6	4.1	31.9
	Chang and Mander^25^	64.3	1.2	51.4	2.6	0.7	4.0	31.6
	No confinement	64.3	1.2	51.4	2.0	0.7	3.1	17.1
Experim	**L**	**53.2**	**1.7**	**42.6**	**2.4**	**1.0**	**2.5**	**25.0**
Anal	Mander et al.^24^	54.5	2.0	–	–	0.8	**–**	**–**
	Kappos et al.^20^	53.4	1.7	42.7	2.4	0.8	3.0	19.8
	Park et al.^21^	53.2	1.7	42.6	2.3	0.8	2.9	17.8
	Chang and Mander^25^	54.1	2.0	–	–	0.8	**–**	**–**
	No confinement	52.1	1.3	41.6	1.8	0.8	2.3	10.4

Experimental values are in bold.

With regard to the maximum force (*F*_*c,max*_), Table 5 shows that all five models predict well the maximum force for all three columns (i.e., C, M and L) with error less than 10%, in line with observations in Figs. 10 and 11. By contrast, the differences between the models and the experimental results are evident for the displacement ductility at ultimate point. For columns C and M, the ductility for the Park et al.^21^, Kappos et al.^20^ and Chang and Mander^25^ models is approximately equal to its experimental counterpart. The ductility for the model with no confinement is notably smaller (i.e., error 12%) than its experimental counterpart for both columns C and M. The observations differ for column L. The ductility for the Park et al.^21^ and Kappos et al.^20^ confinement models, (which are the only two models for which the ultimate point can be determined using the conventions of this study), is notably larger (i.e., error greater than 15%) than its experimental counterpart. By contrast, the ductility for the model without confinement is only 8% smaller than the experimental one.

**Figure 12:**
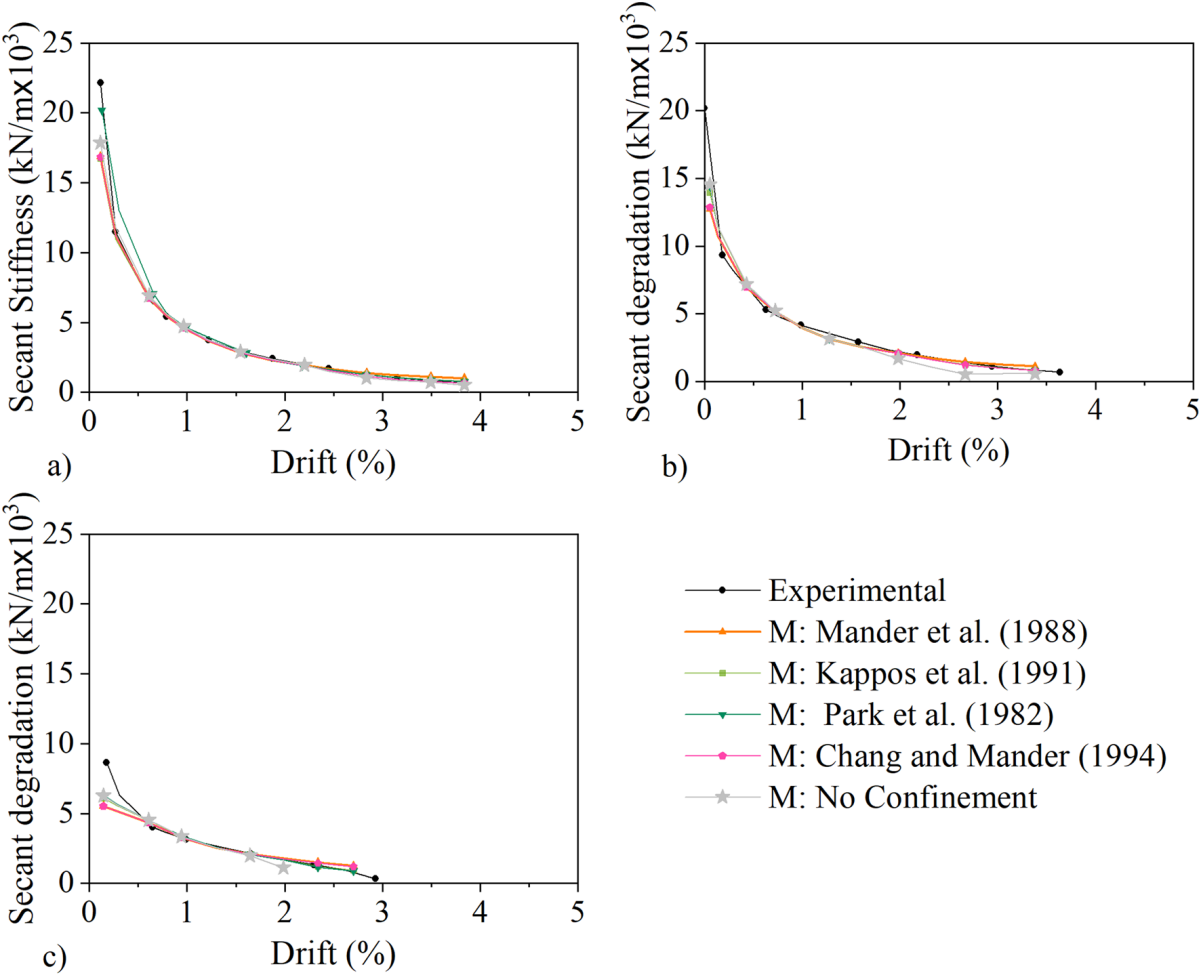
Secant Stiffness: (**a**) column C, (**b**) column M, (**c**) column L.

As observed in Fig. 10, the fire influences the stiffness of the column as it reduces the concrete strength. The difference in stiffness appears to be more pronounced for column L. Ιn Fig. 12, the secant stiffness-drift relationship obtained experimentally and analytically for the five models is presented for the three columns. The secant stiffness is calculated by dividing the maximum compressive force for each cycle with the corresponding displacement. The calculations are based on the positive displacements at each cycle. It should be noted that for all five models the initial secant stiffness obtained analytically is smaller than the experimental one for all three columns. Nonetheless, the differences after 0.5% drift are negligible for all models.

**Figure 13:**
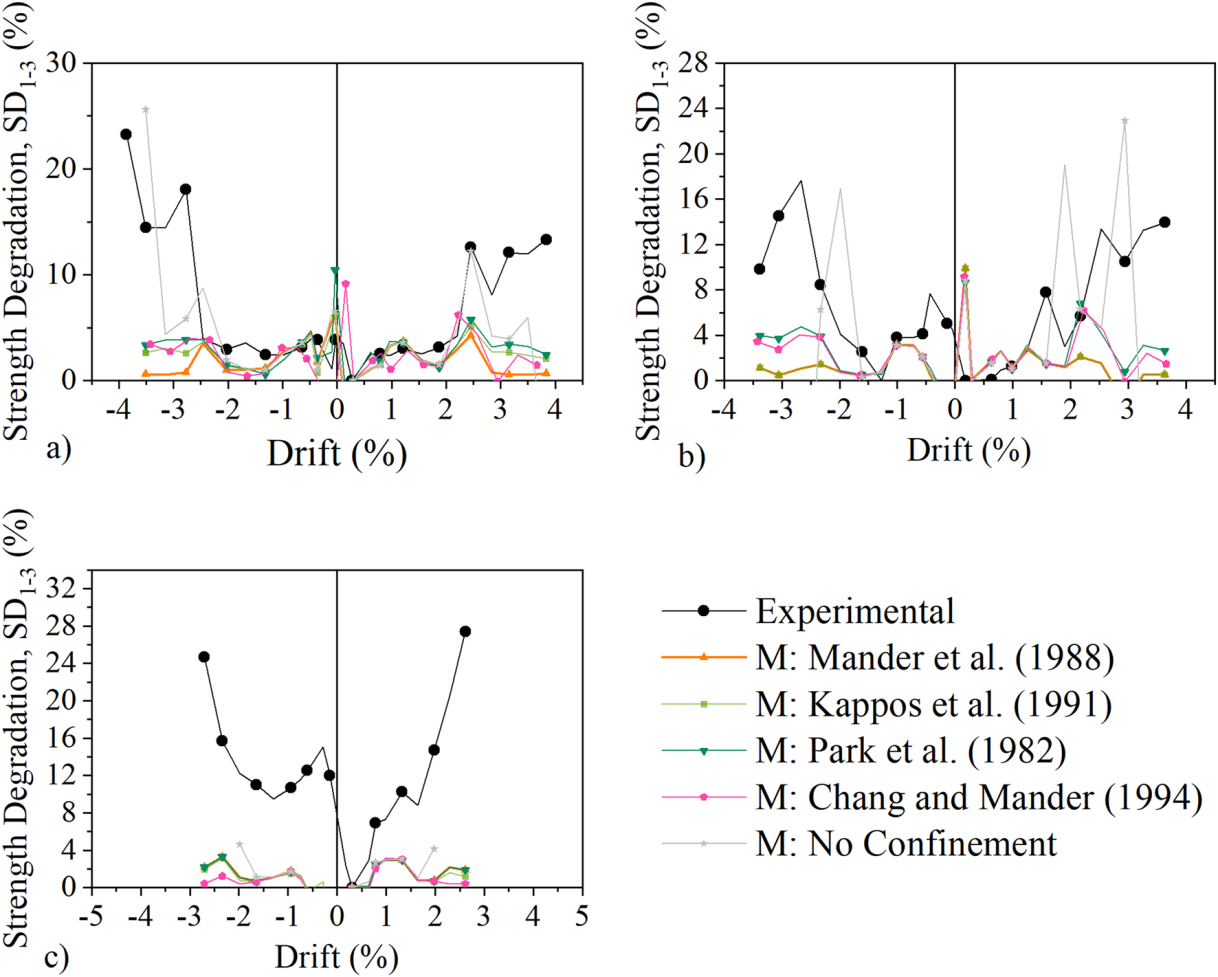
Strength Degradation between 1st and 3rd cycle: (**a**) column C, (**b**) column M, (**c**) column L.

Finally, the strength degradation between the first and third cycle of each drift level obtained experimentally and numerically for the three columns is shown in Fig. 13. It can be seen that for column C, the degradation of strength estimated analytically by all five models follows closely the experimental results up to 2% drift. For the final three cycles associated with larger drifts, the discrepancies are substantial for all models. A similar observation is true for column M. For column L, the discrepancies are substantial for all cycles highlighting the inadequacy of the analytical model to capture the strength degradation within the three cycles of the most severely damaged column.

#### Dissipated energy

**Figure 14:**
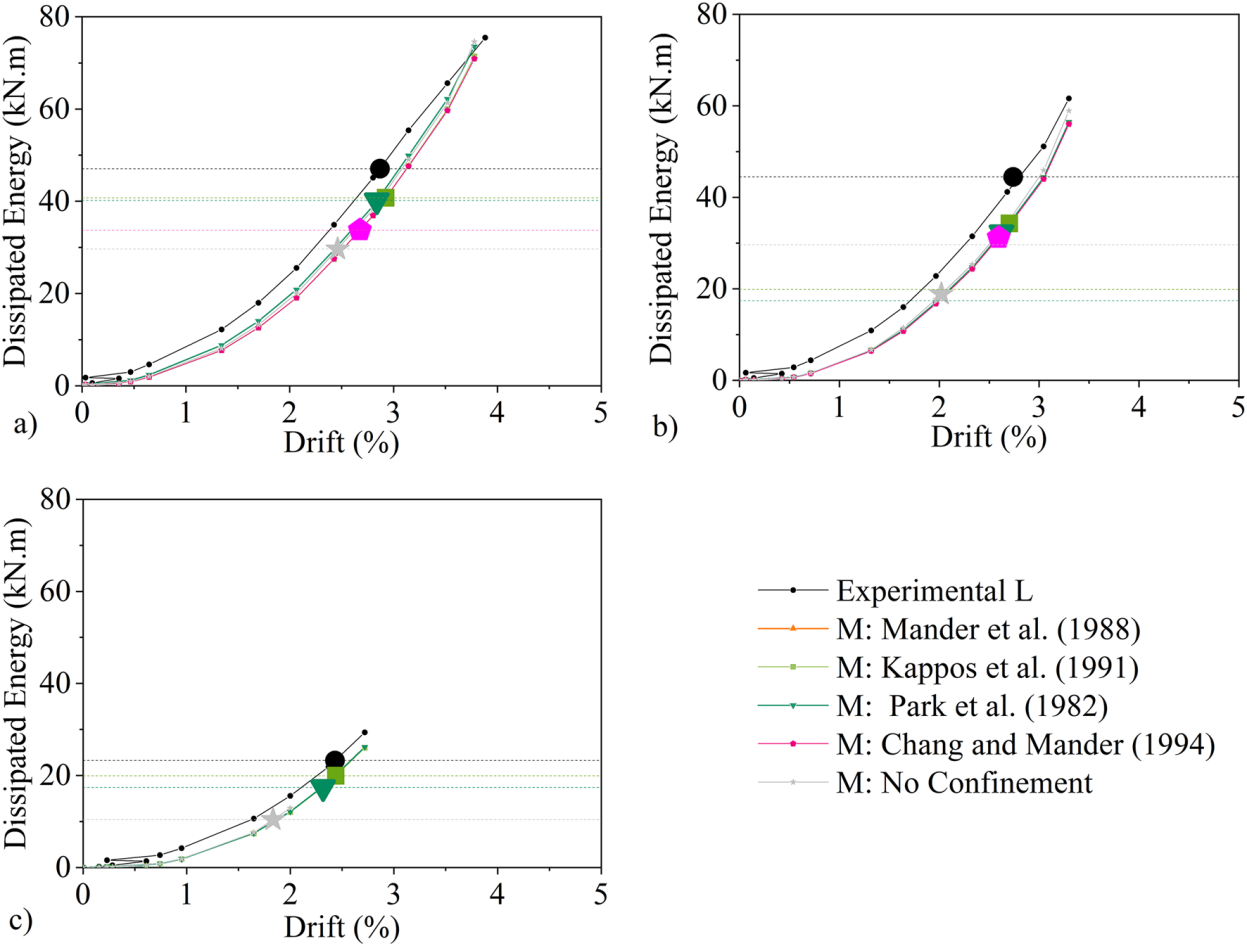
Dissipated energy evolution: (**a**) Experimental Results, (**b**) column C, (**c**) column M, (**d**) column L.

The evolution of the total hysteretic dissipated energy with the drift, based on the experimental and numerical results, is depicted in Fig. 14. The dissipated energy is computed as the sum of the energy dissipation for each hysteretic cycle and corresponds to the interior area of the lateral force–displacement loop of that cycle. The cumulative hysteretic dissipated energy value at the ultimate drift are also distinctly marked in Fig. 14 and their values reported in Table 5. For the experimental results, it is observed that the energy dissipated by columns M and L were respectively 16% and 53% lower that column C up to the ultimate drift. Therefore, the prior fire damage decreases the energy capacity of the columns. When compared to the numerical results, it can be seen that the energy curves overlap substantially and in all cases appear to dissipate less energy than is observed in the experiments.

In Table 5, the values of the cumulative dissipated energy at ultimate drift are reported. Values for the Mander et al.^24^ model for all three columns as well as the Chang and Mander^25^ model for column L are not reported as the ultimate drift could not be estimated as the strength did not deteriorate by 20% for these models. The comparison of the remaining models shows that the Kappos et al.^20^ and Park et al.^21^ models yield very similar results for all three columns. In particular, the energy dissipated by these models for column C is approximately 25% lower that its experimental counterpart. For the other two columns (i.e., M and L), the error from use of the Kappos et al.^20^ remains very similar. However, for Park et al.^21^ models, the error is raised to ~ 30%. By contrast, the no confinement model systematically dissipates substantially less energy than seen in the experiments for all three columns (error equal or larger than 45% for all three scenarios).

Overall, Kappos et al.^20^ model produced analytical results which matched better to the experimental than its alternatives, and Park et al.^21^ followed closely, this justified the recommendation for these two models in the proposed framework in "Framework for modelling post-fire cyclic behaviour of RC column" section.

#### Sensitivity to the mesh used in the heat transfer analysis

The results presented so far are based on heat transfer analysis using a relatively coarse mesh of 8 × 8. In this section, the validity of this is tested by comparing the results with their counterparts based on a finer 16 × 16 mesh. The results of these analyses have been presented in detail in^23^ and a summary of the values of key variables is presented in Table 6. The results for the two meshes are in good agreement (i.e., for most cases the error between the two values is less than 10%) (see Tables 5, 6). This is in line with the literature which focused on estimating the axial capacity of a composite concrete column and suggested that between five^29^ and ten^30^ layers provides accurate results and justifies the recommendation for a coarser mesh in "Framework for modelling post-fire cyclic behaviour of RC column" section.

**Table 6:** Maximum and ultimate lateral force and corresponding drift, drift at yield strength and ductility at ultimate strength (for 16 × 16 mesh).

Source	Column	*F*_*c,max*_	*d*_*c,max*_	*F*_*c,ult*_	*d*_*c,ult*_	*d*_*c*_*,*_*y*_	*μ*_*Δ,ult*_	Diss. energy
		(kN)	(%)	(kN)	(%)	(%)		(kNm)
Experim	**C**	**69.4**	**1.2**	**55.5**	**2.8**	**0.4**	**7.4**	**53.7**
Anal	Mander et al.^24^	68.1	1.2	–	–	0.4	**–**	**–**
	Kappos et al.^20^	68.2	1.2	54.5	2.8	0.4	7.6	40.8
	Park et al.^21^	68.2	1.2	54.6	2.9	0.4	7.8	40.2
	Chang and Mander^25^	68.1	1.2	54.5	2.6	0.4	7.0	33.8
	No confinement	69.8	1.2	55.9	2.4	0.4	6.5	29.7
Experim	**M**	**69.7**	**1.6**	**55.8**	**2.7**	**0.7**	**4.2**	**45.1**
Anal	Mander et al.^24^	63.3	1.2	–	–	0.6	**–**	**–**
	Kappos et al.^20^	63.3	1.2	50.7	2.7	0.6	4.2	33.6
	Park et al.^21^	63.3	1.2	50.6	2.6	0.6	4.1	31.6
	Chang and Mander^25^	63.3	1.2	50.6	2.6	0.7	4.1	32.0
	No confinement	63.1	1.2	50.5	2.0	0.7	3.1	17.2
Experim	**L**	**53.2**	**1.7**	**42.6**	**2.4**	**1.0**	**2.5**	**25.0**
Anal	Mander et al.^24^	53.2	2.0	–	–	0.8	**–**	**–**
	Kappos et al.^20^	52.0	1.7	41.6	2.4	0.8	3.0	19.3
	Park et al.^21^	51.8	1.3	41.4	2.3	0.8	2.9	17.2
	Chang and Mander^25^	53.0	2.0	–	–	0.8	**–**	**–**
	No confinement	50.9	1.3	40.8	1.8	0.8	2.3	10.3

Experimental values are in bold.

## Conclusions

In this study, a framework was proposed on how to construct a simplified model to assess the post-fire performance of a column. The model was validated against the experimental results of a square, non-seismically designed RC column collected as part of the Challenging Risk project in the Structural and Fire Resistance Laboratory of the Aveiro University^2^. Three scenarios are considered. The reference scenario, where the column is exposed only to cyclic loading. In the other two, the column is firstly exposed to an Iso fire for 30 min and 90 min ad after it cools down it is exposed to cyclic loading.

This study concentrated on the sensitivity of the predictions to the confined concrete model used to determine the concrete properties in the core as well as the mesh used to determine the maximum temperature experienced by the cross-section. It was found that a coarser mesh predicts the post-fire cyclic behaviour of the column with very good accuracy. It was also found that the confined model adopted played an important role to describe the behaviour of the column after the peak strength is reached. For the column examined both Kappos et al.^20^ and Park et al.^21^ where found to predict well the overall cyclic behaviour of the column for all three scenarios. By contrast, the Mander et al.^24^ and Chang and Mander^25^ models were found to be unable to account for the post-peak degradation for all three scenarios and only for the L scenario respectively.

## Data Availability

The datasets generated during and/or analysed during the current study are available from the corresponding author on reasonable request.
